# Emerging Roles of Heparanase in Viral Pathogenesis

**DOI:** 10.3390/pathogens6030043

**Published:** 2017-09-18

**Authors:** Neel Thakkar, Tejabhiram Yadavalli, Dinesh Jaishankar, Deepak Shukla

**Affiliations:** 1 Department of Ophthalmology and Visual Sciences, University of Illinois at Chicago, Chicago, IL 60612, USA; neelthakkr511@gmail.com (N.T.); yteja@uic.edu (T.Y.); djaish2@uic.edu (D.J.); 2 Department of Bioengineering, University of Illinois at Chicago, Chicago, IL 60612, USA; 3 Department of Microbiology and Immunology, University of Illinois at Chicago, Chicago, IL 60612, USA

**Keywords:** herpes simplex virus, heparan sulfate, heparanase

## Abstract

Heparan sulfate (HS) is ubiquitously expressed on mammalian cells. It is a polysaccharide that binds growth factors, cytokines, and chemokines, and thereby controls several important physiological functions. Ironically, many human pathogens including viruses interact with it for adherence to host cells. HS functions can be regulated by selective modifications and/or selective cleavage of the sugar chains from the cell surface. In mammals, heparanase (HPSE) is the only known enzyme capable of regulating HS functions via a selective endoglycosidase activity that cleaves polymeric HS chains at internal sites. During homeostasis, HPSE expression and its endoglycosidase activity are tightly regulated; however, under stress conditions, including infection, its expression may be upregulated, which could contribute directly to the onset of several disease pathologies. Here we focus on viral infections exemplified by herpes simplex virus, dengue virus, human papillomavirus, respiratory syncytial virus, adenovirus, hepatitis C virus, and porcine respiratory and reproductive syncytial virus to summarize recent advances in understanding the highly significant, but emerging roles, of the enzyme HPSE in viral infection, spread and pathogenesis.

## 1. Introduction

Heparan sulfate (HS) is a glycosaminoglycan primarily made of repeating disaccharide units of glucosamine and glucuronic acid [1,2]. It is ubiquitously present on cell surfaces and in the extracellular matrix (ECM) often attached to a core protein such as syndecan and/or glypican [1,3]. HS along with its core protein constitute the family of heparan sulfate proteoglycans (HSPG), which regulates many important biological functions crucial for human health and disease. Biosynthesis of HSPG is a complex process requiring the participation of multiple enzymes. The basic HS polymer is composed of alternating glucuronic acid (GlcA) and *N*-acetylglucosamine (GlcNAc) residues. Structural complexity within HS arises from additional modifications, most notably sulfation, and epimerization [4]. Such modifications occur in the Golgi network via the activities of *N*-acetylases, epimerases, and *N*-sulfotransferases. Unique sulfation of glucosamine especially at 2-*O*, 3-*O,* and 6-*O* positions adds structural diversity, which is further enhanced by site specific epimerization of the glucuronic acid residues [1,4,5]. These modifications not only provide structural integrity but also enable HS to take part in affinity-driven cellular processes including direct interactions with various growth factors, chemokines, and cytokines [2]. The modifications also allow for the specificity of functions. For instance, HS with 3-*O* sulfation at glucosamine serves as an antithrombin binding site with an important role in anti-coagulation while 3-*O* sulfated HS generated by a different set of 3-*O* sulfotransferases is responsible for herpes simplex virus (HSV) entry into cells [4,5]. The role of HS is not limited to HSV only as many human and non-human viruses utilize HS as an attachment co-receptor to gain entry into host cells, thus making HS an attractive target for broad-spectrum therapeutic interventions against viral diseases in general [6,7,8,9,10,11,12].

Heparanase (HPSE), a β-d-glucuronidase, is the only known mammalian host enzyme capable of cleaving HS [13]. In 1999 several researchers reported cloning of the HPSE gene. These include Vlodavsky et al. [14], Hulett et al. [15], Kussie et al. [16], Fairbanks et al. [17], and Toyoshima and Nakajima [18]. The gene contains 14 exons and 13 introns and it is located on chromosome 4q21.3 in humans and expressed as two (5 and 1.7 kb) messenger ribonucleic acid (mRNA) transcripts generated via alternative splicing. Another study by Jian et al. [19] provides information regarding the promoter elements on the HPSE gene. In their study, sequence analysis of a GC-rich 3.5 kb HPSE promoter region revealed that the promoter contains two GC boxes and one GT box instead of the usual TATA or CCAAT boxes. It appears that nuclear factor kappa-light chain enhancer of activated B-cell (NF-κB) is among a few known transcription factors that bind the promoter region and control HPSE gene expression [20,21]. Under normal physiological conditions, HPSE expression is higher in placenta and some blood-associated cells, such as mast cells, neutrophils, and lymphocytes. In other human tissues, HPSE expression in a proenzyme form is easily detected but conversion to the active form is tightly regulated [2]. Biosynthesis of native 65 kDa HPSE (proenzyme form) in Golgi apparatus is followed by signal peptide cleavage with the help of cathepsin L. in late endosome/lysosome, resulting in the excision of a 6 kDa peptide, leading to an active heterodimer comprising of 8 kDa and 50 kDa segments [22]. Liquid chromatography-mass spectrometry (LC-MS) experiments conducted by Mao et al. suggested that HPSE cleaves HS at the non-reducing side of highly-sulfated HS domains [23]. HPSE is similar in action to microbial heparinase, an analog of mammalian HPSE, which is classified as three different subtypes (I, II, III), each cleaving HS at a specific site, different from other subtypes [2,24]. An additional isoform of human HPSE, HPSE2, is known but its functions are not well-understood [24].

HPSE regulates the availability of HS chains on ECM or basement membrane (BM) by cleaving HS moieties and thereby controlling various important ligand-binding functions performed by HS [2,23,25]. The sites on HS recognized for cleavage include a specifically-sulfated tetrasaccharide that forms a HPSE binding cleft. The enzyme bound to this cleft cleaves the glycosidic bond between glucuronic acid and *N*-sulfated/6-*O*-sulfated glucosamine or *N*-acetylated/6-*O*-sulfated glucosamine [26,27]. Thus, by regulating the presence of HS moieties on the cell surface, HPSE plays an active role in the tissue distribution of chemokines, growth factors, and lipoproteins. Owing to its unique functionality, studies have suggested important roles for HPSE in cancer metastasis, angiogenesis, inflammation, coagulation and more recently in the stimulation of autophagy and host immune responses [13,25,28].

Keeping in mind the scope of this review article, we searched on public databases including PubMed for recent papers that focused directly on HPSE’s role in viral pathogenesis predominantly in the area of virus-host interaction. Since the area of research that focuses on mammalian HPSE and viral infections is still emerging only a few papers were found. However, it was very clear from the existing literature that HPSE plays an integral role in regulating the lifecycle of many pathogenic human viruses including HSV, dengue virus, human papillomavirus, hepatitis B virus, and hepatitis C virus (Table 1). This review will, therefore, outline the versatile roles of HPSE in virus-host interactions and comment on the future potential of HPSE in effective control of human viral diseases.

## 2. Herpes Simplex Virus (HSV-1) and HPSE

**Figure 1:**
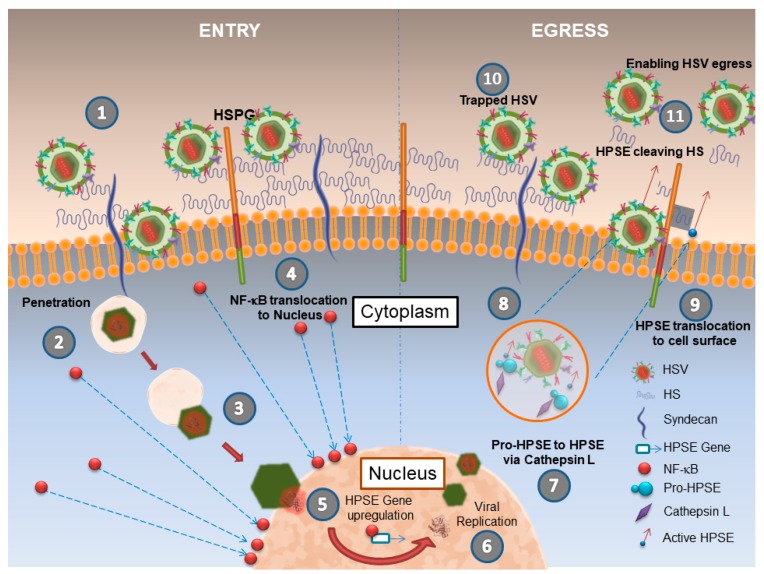
Schematics of HPSE’s role in HSV infection. (1) Virions attach to HSPGs including syndecans on a host cell. (2) Capsid penetration occurs at the plasma membrane (via membrane fusion or endocytosis). (3) The capsid is released into the cytoplasm, which reaches the nuclear membrane for docking and release of viral DNA in the nucleus for replication. (4) Sensing viral invasion, NF-kB translocates to the nucleus and (5) signals HPSE overexpression. (6) New capsids containing viral genomes mature and bud out of the nucleus. (7–8) Enveloped capsids with viral glycoproteins are exocytosed. The same vesicles are also suggested to carry Pro-HPSE, which is eventually activated by cathepsin-L. (9) The enzymatically active HPSE then translocates to the cell surface where it cleaves off heparan sulfate chains (10) to help release any surface bound virions. (11) removal of heparan sulfate enables unrestricted release of HSV-1 virions.

HSV-1 is among the most common human infections. Over 80% of people in the world may be seropositive for this virus, which includes 65% of adults in the United States [29]. HSV-1 can cause mucocutaneous, ocular and systemic illnesses. The virion belongs to the *Alphaherpesviridae* sub-family of herpesviruses, comprising of a lipid coat, a nucleocapsid, tegument proteins, and a double-stranded DNA genome [30,31]. During entry, the HSV-1 virion binds to the cell surface HS via the viral envelope glycoproteins gB and gC, and use this interaction to slide down membrane projections such as filopodia to reach the cell body for membrane penetration [32]. Subsequently, high-affinity binding of gD to one of the many cell surface receptors such as nectin-1, HVEM and 3-*O*-sulfated HS induces conformational changes recruiting gB, gH, and gL for membrane fusion leading to viral penetration and capsid release in the cytoplasm [31]. The capsid then travels to the nucleus where it uncoats at the nuclear membrane and the viral DNA is released in the nucleus for replication. The newly-synthesized capsids containing the re-packaged viral DNA exit the nucleus and ultimately cross the plasma membrane for release of free virions. Hadigal et al. were first to report an important role played by HPSE in the release of newly produced HSV-1 virions (Figure 1) [20]. The authors found that HPSE expression and activity are upregulated in response to HSV-1 infection and as a result, virally-infected cells tend to shed HS from plasma membranes. Due to loss of HS, the infected cells lose the ability to trap any exiting virions which results in cell-free virions that are able to spread to other cells and tissues (Figure 1). This newly-found role of HPSE describes two different modes during infection of a cell by HSV-1. During the attachment mode, virions attach to uninfected cells and enter; whereas, during the detachment mode, the viral progenies cannot attach back to the parental cell; instead they are released to nearby uninfected cells for attachment and infection. This conversion from attachment mode to detachment mode is controlled by upregulation and enhanced the HS-cleaving activity of HPSE. Providing a direct physiological significance and an important role to HPSE in viral pathogenesis, the group found that HSV-1 infection of human corneal epithelial (HCE) cells, which are natural targets for HSV-1 infection, results in higher amounts of enzymatically-active HPSE on cell surfaces and a concurrent loss of HS from the cell surface (Figure 1). The same trend was observed in some other cell-types after infection with additional herpesviruses including cytomegalovirus, bovine herpesvirus, pseudorabies virus, and herpes simplex virus-2. To prove their point further, the authors used a mouse model of corneal infection to demonstrate that transient overexpression of HPSE via a plasmid resulted in an increased virus release and a corresponding decrease in the number of HS moieties on cell surfaces. An opposite trend was observed when HPSE gene was knocked down with shRNA injected directly into the corneas of the animals. In that case, the degree of egress was significantly reduced [20].

Another in vivo study from the same group observed a spike in immune response upon overexpression of GS3-HPSE, a constitutively-active form of HPSE [21]. Corneal overexpression of GS3-HPSE in vivo showed exacerbated herpetic disease characterized by increased infiltration of immune cells and more inflammation. An in vitro wound healing assay using HCE cells overexpressing GS3-HPSE showed delayed healing. A reverse phenomenon was observed when HPSE gene was knocked down by shRNA expression [20]. HPSE was also found to exacerbate disease pathologies and symptoms when overexpressed in corneal cells and tissues due to its ability to elicit inflammation, angiogenesis, and neovascularization [20,28]. As a possible mechanism for enhanced HPSE expression HSV-1 infection was found to upregulate the expression of host factor NF-κB. Upon infection, increased nuclear translocation of NF-κB p65 was observed, which is thought to directly enhance HPSE expression and its promoter activity (Figure 1). It is possible that viral proteins such as infected cell protein 34.5 (ICP 34.5) also regulate HPSE expression and one such possibility was demonstrated by Agelidis et al. [21]. 

## 3. Dengue Virus (DENV) and HPSE

Dengue is the most prevalent arbovirus disease, with the prevalence of 360 million cases worldwide; in which, about 96 million cases manifest to clinical pathology [33]. Systemic plasma leakage causing potentially fatal hypovolemic shock is a major problem among many patients. DENV, part of the Flavivirus family, consists of four serotypes DENV 1–4. DENV particles comprise of a positive-sense RNA genome, capsid, and an envelope encapsulating the capsid [34]. Three structural proteins of Dengue virus have been identified in the past: M, membrane protein; E, envelope protein; and C protein. On the viral envelope M and E protein exist as a heterodimer, however, upon entry into the host, three pH sensitive histidine residues on M proteins are protonated in late endosome causing the dissociation of the heterodimer and exposing the E protein for cellular fusion. E protein has three motifs: DI, DII, DIII; DII is involved in the process of fusion with the host [35]. Unlike herpesviruses, the entire replicative cycle for DENV is completed outside the nucleus.

**Figure 2:**
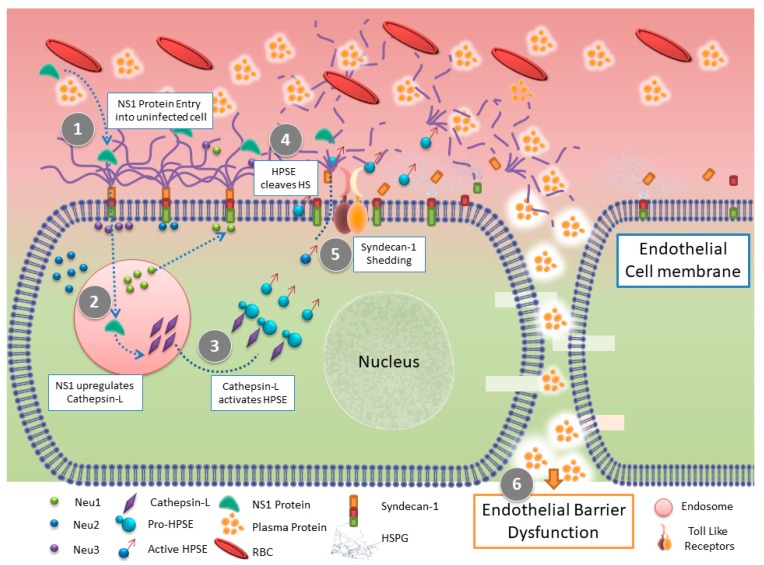
Schematic of HPSE functions in epithelial barrier dysfunction during DENV infection. (1) DENV NS1 protein from an infected cell binds to the surface of an uninfected human pulmonary microvascular endothelial cells (HPMEC) and upregulates the expression and translocation of endothelial sialidases to the cell membrane.(2) DENV NS1 enhances the activity of cathepsin-L and the expression of pro-HPSE. (3) Cathepsin L. activity on pro-HPSE converts it into an active form (4) which leads to the cleavage of heparan sulfate chains on the endothelial glycocalyx layer (EGL) and results in (5) The shedding of Syndecan-1. (6) Puerta-Guardo et al suggest that, together all these processes lead to EGL disruption on endothelial cell surface in-turn resulting in endothelial barrier dysfunction and hyperpermeability that occurs in severe dengue disease.

DENV does not depend on the specific receptor for entry upon attachment; it exploits a variety of host specific receptors to gain entry. Some of the host receptors include glycosaminoglycans such as HS and lectins, an adhesion molecule of dendritic cells, mannose receptors of macrophage, heat shock proteins such as HSP70 and HSP90, and endoplasmic reticulum chaperonin GRP378 [36]. Upon attachment, the virion can be endocytosed by either clathrin-dependent or clathrin-independent macropinocytosis pathways. Puerta-Guardo et al. suggested that HPSE plays a key role during the DENV infection by extracellular glycocalyx layer (EGL) remodeling, causing hyperpermeability, and vascular leakage leading to hypovolemic shock [37]. DENV’s non-structural protein, NS1, the only secreted protein from infected cells, upregulates the activity of cathepsin L. resulting in increased activity of HPSE enzyme in human endothelial cells. HPSE is known to shed GAGs such as HS moieties and enhance remodeling of EGL and ECM leading to hyperpermeability (Figure 2). Internalization of NS1 in the endosome possibly contributes to the activation of cathepsin L. or perhaps its interaction with surface receptor leads to the activation of cathepsin L. leading to HPSE activation and EGL disturbances. NS1 interacts with TLR-4 receptor leading to the activation of intracellular signaling terminating with the upregulation of HPSE. TLR-4 signaling pathways upregulate NF-κB due to its involvement as a downstream effector. This study establishes HPSE as a potential therapeutic target to treat DENV infection.

## 4. Human Papilloma Virus (HPV) and HPSE

Globally, HPV is the most common sexually-transmitted infection. In the US, anogenital HPV infection is common with an annual incidence of 5.5 million infections and an estimated prevalence of 20 million infections. Of many strains known, strains that are sexually transmitted and cause genital warts are also known to cause the cervical cancer pathology. The virus is non-enveloped, contains the capsid, and has a small DNA genome [38]. HPV particles consist of pentamers of prime capsid protein L1, which forms the outer shell of the virus and encapsulates the genome. Additionally, the L2 capsid protein is hidden in the capsid structure with *N*-terminus lying on the capsid surface. HPV16 and HPV18 are the most common strains of the virus known to infect humans and contribute to oncogenesis.

Recent work from Surviladze et al. [39] shows that HPV16 particles bind the ECM via HS chains. Reduction of matrix metalloproteinase and HPSE activities dramatically reduced virus release from the ECM, which resulted in the loss of viral uptake and infection of human keratinocytes. Conversely, exogenous heparinase activated viral release and enhanced infection of keratinocytes. The significance of the authors’ findings may be important, especially at the site of wounds, where the host’s healing response and RTK/GFR signaling upregulate the HS shedding allowing for anoptimal environment for HPV to infect the keratinocytes.

**Figure 3:**
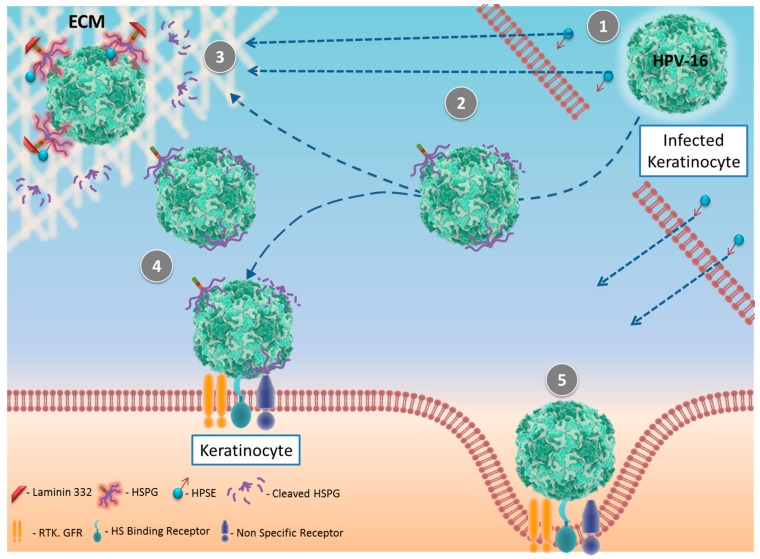
Schematics of HPSE’s role in HPV 16 infection. (1) Mammalian heparanase from surrounding cells or extrinsically added bacterial heparinase III (2) cleaves heparan sulfate proteoglycan (HSPG) chains attached to the surface of HPV16 or (3) reaches the extracellular membrane (ECM) where HPV16 particles attached to the ECM via HSPG are cleaved and released. (4) Released HPV16 viruses are infectious and (5) infect surrounding keratinocytes during a wound healing in vivo model, as shown by Surviladze et al [39].

Further investigative work by Hirshoren et al. showed the significance of HPV E6 gene in HPV-HPSE interaction in head and neck squamous cell carcinoma (HNSCC) [40]. As described in the paper, HPV gene E6 interacts with p53 by downregulating its activity, leading to higher HPSE expression as p53 is a potent inhibitor of HPSE transcription; as shown in Figure 3, p21 expression, a downstream effector of the p53 pathway, positively correlates with HPSE expression in tissue section staining confirming the HPSE-p53 signaling event. Polysaccharide segments of HS moieties serve as attachment sites for many growth factors, cytokines, chemokines, and various bioactive ligands; cleavage of HS by HPSE releases these bioactive factors increasing the invasiveness and malignancy of tumors in the case of HPV16-induced HNSCC.

## 5. Respiratory Syncytial Virus (RSV) and HPSE

RSV is a common infection with nearly all children affected by two years of age. The virus is a member of the *Pneumovirinae* subfamily, derived from *Paramyxoviridae* family [41]. RSV pathology results in symptoms ranging from mild fever and cough to dyspnea and respiratory tract infection (pneumonia). In healthy children and adults the symptoms of RSV infection are mild and typically resemble the common cold. However, in some cases, RSV infection can be severe, especially in premature babies and infants. RSV can also become serious in older adults, adults with heart and lung diseases, or immunocompromised individuals. RSV virion comprises of a nucleocapsid encased in a lipid envelope membrane obtained from the host. The attachment protein G, fusion protein F, matrix protein M, plays an essential role in RSV lifecycle. Additionally, nucleocapsid proteins N, P, L, and M2-1 direct the transcription activity of the genome; M2-2 protein is critical in maintaining the balance in RNA synthesis [31,41,42].

A study conducted by Tao et al. suggested a role of HPSE in RSV-associated neuropathy, leading to proteinuria [43]. HPSE was observed to be upregulated upon RSV infection (RSV4, RSV8, RSV18, RSV19). Expression levels of HPSE mRNA positively correlated with marked proteinuria and urinary protein level, at 24 h post infection; this could be important to the loss of negative charged moieties in the glomerular basement membrane, aiding to the pathogenesis of RSV. Another study by Dong et al. showed the susceptibility of different cell lines to RSV infection by manipulating the surface receptor HS by HPSE [44]. They found that treatment with three bacterial isoforms of heparinase (I, II, III) results in loss of infection. While such treatment may not be possible in humans, especially given the regulatory functions of HPSE in many important physiological pathways, the findings indeed implicate HPSE as a master regulator of RSV infection.

## 6. Adenovirus (ADNV) and HPSE

Adenovirus is a member of Adenoviridae family, consisting of fifty-two distinct serotypes of viruses, categorized into subtype A–F. ADNV is ubiquitous in humans and is endemic throughout the year. It is transmitted via aerosolized droplets, direct inoculation to the conjunctiva, a fecal-oral route, or direct exposure to infected tissue or blood. The virus is capable of infecting many human organs and tissues; however, most infections are asymptomatic. ADNV conjunctivitis is the most common manifestation of human infections by the virus. ADNV virion structure consists of non-enveloped, icosahedral-shaped capsid containing the dsDNA genome, protein, and a trace amount of carbohydrate (fiber protein) [31,45]. ADNV utilizes cellular receptor coxsackie B virus and adenovirus receptor (CAR), located at the tight junctions, for entry upon binding of ADNV fiber protein carboxy terminus with CARS [45,46]. ADNV type B utilizes CD46 receptor expressed on dendritic cells, and serotype D uses sialic acids residues in ECM [46]. There are 11 virion proteins with standard naming II-IX, IIIA, terminal protein and p53 viral protease [31,45]. ADNV genome comprises of five early transcription units (E1A, E1B, E2, E3, E4), three late transcriptional units (IX, Iva2, E2 late) and a later transcriptional unit (L1 to L5). Recently, many research groups have diversified the role of ADNV by using it for gene therapy, virotherapy, and for the treatment of carcinogenesis [47,48,49].

Investigative work from Watanabe et al. used HPSE in virotherapy for human malignant pleural mesothelioma [50]. Development of attenuated, replication-selective, adenovirus 5 vector (OB-301) with human telomerase reverse transcriptase promoter, which drives the expression of E1A and E1B genes linked with internal ribosome entry, delivered via intrapleural administration, resulted in the selective killing of tumor cells. Administration of HPSE expressing adenovirus (AD/hep) along with OB-301 enhanced the efficacy of virotherapy by reducing the hindrance of ECM. AD/hep coinfection with OB-301substaintially reduced the ECM hindrance by expression of HPSE; this resulted in substantial tumor weight reduction along with deeper virus penetration into tumor spheroid. Indeed, HPSE is known to induce expression of MMPs, which further increased the efficacy of virotherapy. Perhaps, HPSE is known to exacerbate cancer by increasing tumor metastasis, however, the experimental dosage of the combo did not show any tumor malignancy, suggesting its use to be safe for therapeutic purposes.

## 7. Hepatitis C Virus (HCV) and HPSE

HCV, a distinct serotype from HAV and HBV, belongs to the *Hepacivirus* genus under Flaviviridae family [31]. It is a blood borne virus that causes both acute and chronic hepatitis, with multiple severity levels, from a mild illness that only lasts a few weeks to a more serious, lifelong illness. HCV transmission can occur through exposure to infected blood usually during injected drug use, blood transfusion of unscreened blood and unsafe use of surgical instruments. Globally, about 71 million people have chronic HCV infection. A significant number of chronically infected individuals develop liver cirrhosis or hepatocellular carcinoma. HCV contains positive-stranded RNA genome encased in nucleocapsid surrounded by lipid membrane envelope [51]. HCV virus positive sense RNA genome encodes for a polyprotein of approximately 3000 residues flanked by small 5′ and 3′ untranslated region [52]. The polyprotein comprises of three portions: contains an amino segment that contains structural protein (core, E1, E2); a central region that includes for protein p7 and NS2, potentially involved in viral morphogenesis and egress; and carboxyl end segment includes non-structural proteins (NS3, NS4A, NS4B, NS5A, NS5B) [52]. This lengthy polyprotein is co- and post-translationally modified by host and viral proteases before packing into the nucleocapsid. HCV infection perplexed clinical pathology includes hepatocellular carcinoma, hepatitis, and potentially cirrhosis [53]. 

Investigative work has reported the utilization of a HSPG cell surface receptor by HCV virion for attachment to the host [54]. El-Assal et al. noted a significant increase in HPSE expression in HCV-positive HCV patient compared to HCV-negative patient indicating a possible role of HPSE in exacerbating the pathology caused by HCV [55]. While the exact significance of their findings is not completely clear, their findings do implicate HPSE in progression of chronic infections, which needs to be further examined. 

## 8. Porcine Respiratory and Reproductive Syncytial Virus (PRRSV) and HPSE

PRRSV belongs to *Arteriviridae* family, order *Nidvirale* [31]. PRRSV, a porcine virus, causes a panzootic disease, which results in reproductive failure in breeding stock and respiratory illnesses in young pigs. PRRSV is an enveloped virus encapsulating the capsid comprising of positive sense RNA genome [56]. PRRSV envelope consists of gp2, gp3, gp4 glycoproteins, envelope protein (E), and membrane protein (M), aiding in the process of attachment and host invasion. The principal component of the capsid is N protein (nucleocapsid protein), which has been implicated in localizing viral protein assembly in nucleolus; indeed, N protein has been implicating in the regulation of viral gene transcription by transcribing the RNA-based RNA polymerase [57].

Guo et al. from China recently published a paper depicting the role of HPSE in PRRSV release from cells [58]. The findings were similar to those reported by Hadigal et al. using herpesviruses [20]. The authors, in a separate paper, also demonstrated that HPSE can be a potent target for pyrithione (PT) [59]. PT inhibits the PRRSV pathogenesis in porcine alveolar macrophages (PAM) cells and marc-145 cell line potentially via increased influx of Zn^2+^ ions, which may, in turn, negatively impact HPSE activity. As claimed in the article, PRRSV infection results in a surge of nuclear localization of host factor NF-κB leading to the upregulation of p65 transcription factor, eventually increasing the expression of HPSE. PT treatment inhibits the localization of p65 transcription factor into nucleus reducing the HPSE expression.

## 9. Future Therapeutic Potential of Targeting HPSE in Viral Diseases

Several HPSE inhibitors have entered clinical trials for various cancers but none yet for viral diseases. These include Muparfostat (PI-88), Roneparstat (SST0001), PG545, and necuparanib (M402) [60]. Initial findings already suggest that targeting HPSE can have rewarding benefits in controlling many viral diseases and, therefore, the above-mentioned drugs or their analogs may be highly beneficial against viral diseases. For herpesviruses, HPSE appears to function in a fashion similar to influenza virus neuraminidase, which removes sialic acid during the viral egress. Various research labs have utilized this relationship to develop new therapies against influenza virus [61]. A similar approach with focus on HPSE-HS interaction could also provide a promising route for new and more effective therapeutic interventions against herpesviruses. In support of this possibility, pharmacological treatment of ex vivo porcine corneas with a known HPSE inhibitor, OGT 2115, decreased viral release and associated pathologies [20]. The results were impressive promising a novel therapeutic intervention against herpesviruses, which cause numerous health and social problems. A similar benefit was also proposed for the control of hypovolemic shock after DENV infection. As demonstrated by Puerta-Guardo et al. [37] treatment of DENV infected human pulmonary microvascular endothelial cells with OGT 2115 significantly reduced the EGL disruption and brought the transendothelial electric resistance back to normal. A similar targeting of HPSE is likely to have strong health benefits against the other viruses discussed in this review, including PRRSV. Currently, there is no effective treatment for PRRSV. Broad-spectrum antibiotics may control secondary infections and anti-inflammatory drugs (e.g., aspirin) can be administered during acute disease. Now the promise exists that targeting HPSE or an upstream effector, p65, could provide a new therapeutic intervention to treat the disease. Overall, the emerging knowledge on HPSE as an important regulator of viral infections and associated morbidities could one day make a broad-spectrum antiviral drug a real possibility.

## 10. Future Prospects and Conclusions

Heparanase was already implicated in inflammation, angiogenesis, and cancer progression and over the past few years significant new progress has been made in elucidating some unique functions of HPSE in viral infection and dissemination. Notably, emerging data show that in addition to its well characterized role in cancer, HPSE activity may represent an important determinant in the pathogenesis of several viral diseases, including those caused by HSV, DENV, ADNV, HPV, HCV, and some retroviruses (Stavrou, Agelidis, and Shukla, unpublished results). It is interesting that many of the viruses that depend on HPSE for infectivity also cause cancers, such as HPV and HCV. Thus, it is possible that a future antiviral drug may be able to simultaneously control both: viral growth and cancer progression. According to the available data and experimental evidences, HPSE will surely have multiple well-defined roles in several fields spanning from prognosis to diagnosis and prevention to therapy of viral and non-viral diseases. In this regard newer and more effective strategies aimed at blocking HPSE activity, identification of novel receptors mediating its signaling-related activities, and a better understanding of its functions in the cell nucleus will certainly advance our knowledge of HPSE in health and disease. Moreover, the expected positive results of ongoing clinical trials using HPSE inhibitors will also shed more light on the advantages (or drawbacks) of developing HPSE as a therapeutic target.

## Figures and Tables

**Table 1:** Important roles for HPSE in viral infections.

Virus	Role of Viral Protein in Pathogenesis (Direct/Indirect)	Role of HPSE in Pathogenesis	References
**HSV-1**	Direct	Increased virus release, ECM damage leading to disease pathologies	[20,21]
**DENV**	Indirect	Increased in severity of disease symptoms	[37]
**HPV**	Direct	Increased virus release	[39]
**RSV**	Direct	Increased rate of infection	[43,44]
**ADNV**	Indirect	Increased rate of infection, virotherapy of cancers	[47–49]
**HCV**	Not determined	Not determined	[54]
**PRRSV**	Direct	Increased virus release	[58,59]
